# Book Reviews

**Published:** 1894-09

**Authors:** 


### Book Reviews.

CLINICAL GYNECOLOGY: BEING A HAND-BOOK OF DISEASES PECULIAR TO WOMEN. By THOMAS MORE MADDEN, M. D., F. R. C. S., Ed., Obstetric Physician and Gynecologist, Mater Misericordiæ Hospital, Dublin; Consulting Physician, Hospital for Sick Children, etc., etc. Octavo, pp. xvi.—562, with 259 illustrations. Philadelphia: J. B. Lippincott Company. 1893.

Madden has been for many years a recognized authority on diseases of women, consequently what he has to offer always merits and receives the patient attention of the profession of medicine.

This book comprises the lectures delivered by the author to his hospital class and his contributions to medical journals. They have, therefore, the stamp of personality upon them, and are published in compliance with oft-repeated requests on the part of his pupils and confrères. Their object, therefore, is to impart a clinical knowledge which the author asserts has oftentimes been acquired in a slow manner and frequently has been the result of dearly bought experience.

Madden divides his book into eight parts. In Part I., consisting of ten lectures, he deals with diseases and injuries of the perineum, vulva, bladder and vagina. Lecture second is devoted to a consideration of the methods of ordinary gynecological investigation. These are the methods usually described in treatises on gynecology, but Madden, we think, lays too much stress on the value of the uterine sound. He begins by recognizing the harmfulness of the instrument, but regards the sound as no less necessary to the gynecologist than the stethoscope or clinical thermometer is to the physician. In view of the fact that the uterine sound is being discarded as a useless instrument by the majority of gynecologists, we think this, to say the least, is pretty strong language.

Diseases and abnormalities of the uterus are treated upon in Part II., which comprises eleven lectures. These are, for the most part, well up to the modern standard in consideration and treatment.

Uterine displacements and flexions occupy Part III., consisting of six lectures. In this the usual number of pessaries are illustrated, most of which are of no use whatever.

In Part IV., devoted to consideration of diseases of the uterine appendages, Madden becomes interesting, and the eight lectures here given are full of instruction. The author, however, for the most part, follows the plans laid down by other treatises, and borrows many of their illustrations, always, however, with credit.

The remaining parts, in their consecutive order, treat of menstruation and its disorders; constitutional disorders connected with menstruation; pelvic inflammation and hematocele, and the diseases and abnormalities of pregnancy.

While this treatise has very many valuable points and is the result of patient observation by a careful teacher, yet we are not impressed with the fact that there is a great necessity for its appearance. It will be sought eagerly, however, by gynecologists, who must necessarily possess all literature on the subject that is published, especially when by such able authors as the present.

---

INTERNATIONAL CLINICS. A Quarterly of Clinical Lectures on Medicine, Neurology, Pediatrics, Surgery, Genito-Urinary Surgery, Gynecology, Ophthalmology, Laryngology, Otology and Dermatology. By professors and lecturers in the leading medical colleges of the United States, Great Britain and Canada Edited by JUDSON DALAND, M. D., Philadelphia, Instructor in Clinical Medicine and Lecturer on Physical Diagnosis in the University of Pennsylvania ; Assistant Physician to the University Hospital ; Physician to the Philadelphia Hospital and to the Rush Hospital for Consumption. J. Mitchell Bruce, M. D., F. R. C. P., London, England ; Physician and Lecturer on Therapeutics at the Charing Cross Hospital. David W. Finlay, M. D., F. R. C. P., Aberdeen, Scotland ; Professor of Practice of Medicine in the University of Aberdeen, Physician to, and Lecturer on, Clinical Medicine in the Aberdeen Royal Infirmary ; Consulting Physician to the Royal Hospital for Diseases of the Chest, London. Volume I., Fourth series, 1894. Royal 8vo, pp. xii.—363. Philadelphia : J. B. Lippincott Co. 1894.

This volume opens with a memorial of Prof. Jean-Marie Charcot, by Dr. M. Allen Starr. Charcot's eminence as a clinician and a scientist fully entitle him to the words of praise bestowed by the memorialist. It is accompanied by a medallion etching in profile of Charcot, published as a frontispiece.

The local contributors to this volume are Drs. Charles G. Stockton, on Amebic Dysentery ; Dr. Roswell Park, on Macewen's Operation for Rachitic Deformity of the Femur—Inflamed Hemorrhoids ; and Dr. Matthew D. Mann, on Ruptured Tubal Pregnancy.

These clinics continue on the same popular lines as heretofore and are being received with increasing favor by the medical profession.

---

REPORT OF THE COMMISSIONER OF EDUCATION FOR THE YEAR 1890-91. Volumes I. and II. Washington : Government Printing Office. 1894.

This report, although three years behind time, contains much of interest to educators. We think that such important questions as are evolved under the general term, education, deserve to be clothed in better manner than are these books. The government should not apply the same rules as to paper and binding to reports on education that it does to the patent office reports ; and, again, it should print the education reports more promptly. These are intended to become a part of the various libraries of the land, and should be printed on good paper and bound in a substantial manner.

Essentials of Anatomy, Including the Anatomy of the Viscera. Arranged in the form of Questions and Answers ; prepared especially for Students of Medicine. By Charles B. Nancrede, M. D., Professor of Surgery and of Clinical Surgery in the University of Michigan, Ann Arbor; Corresponding Member of the Royal Academy of Medicine, Rome, Italy, etc., etc. Saunders' Question Compends, No. 3. Fifth edition, with an appendix and 180 illustrations. Duodecimo, pp. x.—388. Price, $1.00. Philadelphia: W. B. Saunders, 925 Walnut street. 1894.

Essentials of Nervous Diseases and Insanity. Their Symptoms and Treatment. By John C. Shaw, M. D., Clinical Professor of Diseases of the Mind and Nervous System, Long Island College Hospital Medical School ; Consulting Neurologist to St. Catharine's Hospital and Long Island College Hospital. Saunders' Question Compends, No. 21. Second edition. Crown 8vo, 186 pages. Forty-eight original illustrations, mostly selected from the author's private practice. Price, cloth, $1.00 ; interleaved for notes, $1.25. Philadelphia : W. B. Saunders, 925 Walnut street. 1894.

The first of these books, the Essentials of Anatomy, by Nancrede, having already passed to its fifth edition, its status is already determined. As a teacher of anatomy, Nancrede has few equals. Anatomy, too, is a subject for the memory to grasp, and such books as this which especially contribute to aid the memory are not only admissible but valuable.

The second book, The Essentials of Nervous Diseases and Insanity, is an exceedingly well planned condensation of the subject, but it has not the same place that the anatomical essentials possess, because the subject is not one to be memorized in the same sense that is anatomy. However, the author has made the most of his opportunity, and the book serves to make one of a very useful series.

---

### BOOKS RECEIVED.

Annual Report of the State Board of Charities for the year 1893. Albany: James B. Lyon, State Printer. 1894.

The Johns Hopkins Hospital Reports. Volume IV., Nos. 4-5. Report in Neurology II. Baltimore : The Johns Hopkins Press. 1894.

Transactions of the Association of American Physicians. Ninth session, held at Washington, D. C., May 29, 30, 31, and June 1, 1894. Philadelphia : Printed for the Association. 1894.

International Clinics. A Quarterly of Clinical Lectures on Medicine, Neurology, Pediatrics, Surgery, Genito-Urinary Surgery, Gynecology, Obstetrics, Ophthalmology, Laryngology, Otology and Dermatology. By professors and lecturers in the leading medical colleges of the United States, Great Britain and Canada. Edited by Judson Daland, M. D., Philadelphia, Instructor in Clinical Medicine and Lecturer on Physical Diagnosis in the University of Pennsylvania ; Assistant Physician to the University Hospital ; Physician to the Philadelphia Hospital and to the Rush Hospital for Consumptives. J. Mitchell Bruce, M. D., F. R. C. P., London, England, Physician and Lecturer on Therapeutics at the Charing Cross Hospital. David W. Finlay, M. D., F. R. C. P., Aberdeen, Scotland, Professor of Practice of Medicine in the University of Aberdeen; Physician to, and Lecturer on, Clinical Medicine in the Aberdeen Royal Infirmary; Consulting Physician to the Royal Hospital for Diseases of the Chest, London. Volume II. Fourth series. 1894. Royal 8vo, pp. xii.—363. Philadelphia: J. B. Lippincott Co. 1894.

A System of Genito-Urinary Diseases, Syphilology and Dermatology. By various authors. Edited by Prince A. Morrow, A. M., M. D., Clinical Professor of Genito Urinary Diseases; formerly Lecturer on Dermatology in the University of the City of New York; Surgeon to the Charity Hospital, etc. With illustrations. In three large 8vo volumes. Volume III. Dermatology. Pp. xiv.—976. New York: D. Appleton & Co. 1893. Sold only by subscription.

